# Epigenetic Regulators Involved in Osteoclast Differentiation

**DOI:** 10.3390/ijms21197080

**Published:** 2020-09-25

**Authors:** Kristina Astleford, Emily Campbell, Andrew Norton, Kim C. Mansky

**Affiliations:** Department of Developmental and Surgical Sciences, School of Dentistry, University of Minnesota, Minneapolis, MN 55455, USA; astl0008@umn.edu (K.A.); camp0755@umn.edu (E.C.); norto263@umn.edu (A.N.)

**Keywords:** methylation, demethylation, acetylation, deacetylation, osteoclasts, epigenetics

## Abstract

Age related changes to the skeleton, such as osteoporosis, increase the risk of fracture and morbidity in the elderly population. In osteoporosis, bone remodeling becomes unbalanced with an increase in bone resorption and a decrease in bone formation. Osteoclasts are large multinucleated cells that secrete acid and proteases to degrade and resorb bone. Understanding the molecular mechanisms that regulate osteoclast differentiation and activity will provide insight as to how hyper-active osteoclasts lead to pathological bone loss, contributing to diseases such as osteoporosis. Reversible modifications to the DNA such as histone acetylation, methylation, phosphorylation and ubiquitylation alters the access of transcriptional machinery to DNA and regulates gene expression and osteoclast differentiation and activity. It is critical for the management of bone related diseases to understand the role of these chromatin modifying proteins during osteoclast differentiation, as potential therapies targeting these proteins are currently under development.

## 1. Introduction and Osteoclast Biology

Osteoclasts are large multinucleated cells of hematopoietic origin that degrade the bone matrix. They are formed by fusion of mononuclear precursors of the monocyte/macrophage lineage, and are the primary resorptive cells of the skeleton [1,2]. Progression from multipotent progenitors into specialized, terminally differentiated cells involves carefully regulated patterns of gene expression to control lineage specification and emergence of the cellular phenotype. This process requires coordinated action of transcription factors with co-activators and co-repressors to bring about proper activation and inhibition of gene expression.

**Figure 1:**
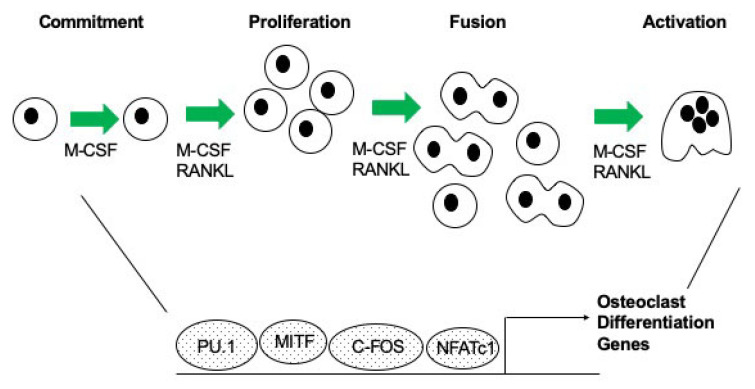
Image depicting stages of osteoclast differentiation and the complex of transcription factors that regulate gene expression.

Osteoclast gene expression is activated by a group of transcription factors. It has been demonstrated through multiple studies that c-FOS, PU.1, MITF and NFATc1 act in concert to regulate genes necessary for osteoclast differentiation (reviewed in [3], Figure 1). The following is a brief description of what is known about individual transcription factors, and their role in regulating osteoclast differentiation. PU.1 is a member of the ETS-domain transcription factor family [4]. It has been shown that mice lacking PU.1 have a deficiency in both macrophage and osteoclast differentiation, and this established PU.1 as one of the earliest markers of the osteoclast lineage [5]. Microphthalmia transcription factor (MITF) is a basic helix-loop-helix-leucine zipper transcription factor [6,7,8]. It has been shown that MITF plays an essential role in regulating gene expression during osteoclast differentiation. C/EBPα has been shown to be important for differentiation of myeloid progenitors [9]. Recently, it was determined that C/EBPα is highly expressed in osteoclasts [10]. c-FOS is a member of the activator protein-1 (AP-1) family of transcription factors, and its expression is induced early during osteoclast differentiation [11,12]. c-FOS acts as an important switch between osteoclast and macrophage differentiation, and in its absence, osteoclasts do not form [11]. Nuclear factor kappa-light-chain-enhancer of activated B cells or NF-κB is a pleiotropic transcription factor and is part of the Rel subfamily of proteins [13]. In osteoclasts, NF-κB regulates formation, function and survival [13,14,15]. NF-κB is activated downstream of RANK signaling that ultimately results in the activation of NFATc1 to induce osteoclastogenesis [13,16]. NFATc1, the master regulator of osteoclast differentiation, mediates RANKL-induced osteoclast formation, and its overexpression in *c-Fos* deficient cells rescues osteoclastogenesis [12,17].

Epigenetics are external modifications that do not change the DNA sequence but none the less regulate expression of target genes. Understanding epigenetic modifiers in osteoclasts has recently become a major focus in understanding how these cells are transcriptionally regulated during aging and osteolytic diseases. Epigenetic mechanisms discussed in this review include histone modifications (Section 2), DNA/RNA methylation and noncoding RNAs (Section 3). Post translational modifications (PTMs) of histones result in changes in chromatin arrangement and subsequent gene transcription. There are three main types of proteins that play an important role in the epigenetic regulation of gene transcription: “writers”, “erasers” and “readers”. “Writers” are proteins that are responsible for adding histone modifications to histone tails to either activate or repress gene transcription. The main types of modifications that can be added to histone residues are acetylation, methylation, ubiquitination and phosphorylation. Contrary to “writers”, “erasers” are proteins that remove histone modifications to regulate transcription. Lastly, “reader” proteins can bind to histone modifications to simply identify and/or recognize what type of modification is present at the location. In the following sections, we will describe what is currently known about epigenetic proteins that regulate osteoclast differentiation.

## 2. Epigenetic Regulation by Histone Modifications

Methylation and acetylation are the two most common types of modifications to histones. In Section 2.1 and Section 2.2, we will discuss what is known in osteoclasts about epigenetic regulators that add methyl or acetyl groups to histones. These are the “writers”. Section 2.3 and Section 2.4 will discuss the “erasers” and the information that is known about epigenetic regulators in osteoclasts that remove methyl and acetyl groups from histones.

### 2.1. Histone Methyltransferases

**Figure 2:**
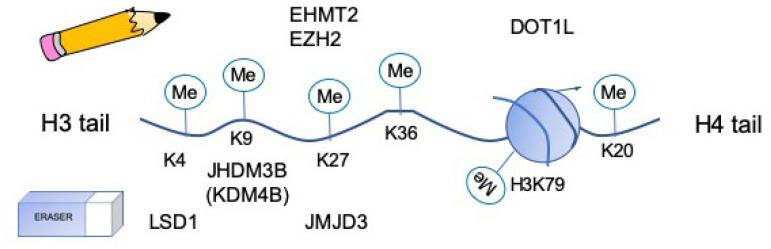
Schematic of location of lysine residues methylated by methylases (“writers”) and demethylases (“erasers”) that have been shown to regulate osteoclast differentiation.

The major role of histone methyltransferases is to add methyl groups to histone tail residues. There can be up to 3 methyl groups added to a residue at a time, making the residue either mono-, di- or trimethylated (Figure 2, [18]). Methylation occurs on basic residues such as lysine and arginine and either leads to transcriptional activation or repression, depending on the residues targeted. Histone methyltransferases are divided into three groups: SET domain containing lysine methyltransferases, DOT1- like lysine methyltransferases and Protein Arginine N-methyltransferase (PRMT) family.

#### 2.1.1. *Ehmt2*

Euchromatic histone-lysine N-methyltransferase 2 (also known as EHMT2, G9a and H3K9me1.2 methyltransferase) is a member of the SET domain containing lysine methyltransferase family. It acts by catalyzing the mono-methylation of H3K27, which is necessary for MMP9-dependent H3NT proteolysis [19]. Selective inhibition of G9a mediated H3K27 methylation completely inhibited RANKL induced osteoclast differentiation and gene expression [19]. Localization patterns of MMP9 and H3K27 mono-methylation were similar in osteoclast target genes, indicating that H3K27me1 was a recruitment signal for MMP9 [19]. As expected, there was no change in H3K27me1 levels when osteoclasts were treated with an MMP9 inhibitor [19].

#### 2.1.2. *Ezh2*

Enhancer of zeste homolog 2 (EZH2) is a component of PRC2 and is also a member of the SET domain containing lysine methyltransferase family. It functions by catalyzing the trimethylation of H3K27 [20]. The use of an EZH2-specific inhibitor demonstrated that EZH2 plays a role in promoting osteoclast differentiation by enabling the RANKL-induced expression of *Nfatc1* [20]. Both inhibitor and knock down studies suggest that EZH2 plays a role in osteoclast differentiation during the first 24 h of RANKL stimulation [21]. Further, the catalytic activity of EZH2 directly represses negative regulators *Irf8, MafB,* and *Arg1* by being recruited to the promoters during the early stages of RANKL treatment [20,21]. EZH2 is also found in the cytoplasm of osteoclasts and has been shown to regulate PI3K-AKT-mTOR signaling and cytoskeletal dynamics [21].

#### 2.1.3. *Dot1l*

Disruptor of telomeric silencing 1-like protein (DOT1L) is a member of the DOT1-like lysine methyltransferase family. It acts by catalyzing the di-methylation of H3K79 [22]. Di-methylation of H3K79 was shown to be accompanied by an upregulation in *Nfatc1*, *Acp5* and *Ctsk* and regulates the resorptive activity of RANKL-induced osteoclasts [22]. Inhibition of DOT1L caused a decrease in H3K79me2 and an increase in osteoclast size, surface area and resorptive activity [22]. Not surprisingly OVX animals treated with DOT1L inhibitor had greater losses of bone compared to sham treated animals [22]. While this study demonstrates intriguing results, experiments were performed in RAW 264.7 cells and should be confirmed in primary cells and mouse models.

#### 2.1.4. *Prmt1*

PRMT1 expression is increased with RANKL treatment and osteoclasts from *Prmt1^+/−^* mice were smaller and had impaired F-actin ring formation and bone resorption [23]. PRMT1 was shown to interact with and regulate the transcriptional activity of NF-κB [23]. Lastly estrogen was shown to downregulate expression of *Prmt1*, which suggests that estrogen may negatively regulate osteoclast differentiation by modulating the expression of *Prmt1* [23].

#### 2.1.5. *Prmt5*

Similar to PRMT1, PRMT5 expression increases during osteoclast differentiation [24]. Reduction in expression of PRMT5 by siRNA in osteoclasts inhibited RANKL mediated osteoclast differentiation [24]. PRMT5 inhibitor EPZ015666 suppressed osteoclast differentiation and activity and as expected protected against OVX induced bone loss [24]. PRMT5 inhibition resulted in a decrease in H3R8 and/or H4R3 methylation at the *Cxcl10* and *Rsad2* promoters [24]. This data suggests a possible mechanism for PRMT5 regulation of osteoclast differentiation, especially in diseases such as arthritis where CXCL10 has been shown to play a role in regulating osteoclast activity [24].

### 2.2. Histone Acetyltransferases

Similar to histone methyltransferases, histone acetyltransferases (HATs) are also “writer” proteins that add acetyl groups to histones tails. Additionally, these enzymes also have the ability to acetylate non-histone proteins to regulate their function [25]. Hyperacetylated histones are recognized as a hallmark of transcriptionally active chromatin and therefore acetylation typically enhances gene expression. Mammalian HATs include GNC5, and its ortholog PCAF, CREB-binding protein (CBP), p300 and TAFII250.

#### 2.2.1. *Pcaf*

P300/CBP-binding protein (PCAF) is a transcriptional co-activator that is able to acetylate histones and non-histone proteins such as SMADs, NF-κB and p53 [25]. PCAF HAT activity can be increased through acetylation of its lysine residues by either auto-acetylation or by p300. In osteoclasts, PCAF functions by physically interacting with NFATc1 to acetylate and activate it through RANKL signaling [25].

#### 2.2.2. *p300*

P300 acts as a traditional histone acetyltransferase by activating transcription via histone or non-histone protein acetylation in conjunction with its binding partner CREB-binding protein (CBP). In osteoclasts, p300 has been shown to interact and be activated by phosphorylated MITF and TFE3 to promote expression of osteoclast genes [26]. Additionally, as mentioned above, p300 can acetylate PCAF to enhance its HAT activity [25].

### 2.3. Histone Demethylases

The main function of histone demethylases is to remove methyl groups from histone tails to either repress or activate gene transcription, depending on the residues that are targeted. In most cases, histone demethylases target lysine residues on histone 3 and 4 (Figure 2). The very first histone lysine demethylase discovered was lysine specific demethylase 1 (LSD1), also known as KDM1A, and since then, 16 histone demethylases have been identified [18,27]. Histone demethylases are known to be conserved throughout species and many times these demethylases have redundant functions [18]. Additionally, many of these proteins are coordinated in their activity and share substrate specificity [18]. Currently, there is not much known about how many of these demethylases function in osteoclasts, however LSD1, KDM4B and JMJD3 have been shown to be important in osteoclast differentiation.

#### 2.3.1. *Lsd1*

Lysine specific demethylase (LSD1 or KDM1A) is a FAD+ dependent protein enzyme that is known to demethylate mono- and di- methyl histone 3 lysine 4 and 9 (H3K4 and H3K9, respectively). Demethylation of H3K4 results in gene repression and demethylation of H3K9 results in gene activation. Currently, preliminary data from the Mansky lab has shown that inhibition and knockdown of LSD1 enhances osteoclast differentiation, suggesting that LSD1 acts as a transcriptional repressor in osteoclasts. This is supported by an increase in mono-methylation at H3K4 but no change in methylation at H3K9 (Mansky, unpublished observation). The mechanism by which LSD1 functions is currently being investigated.

#### 2.3.2. *Kdm4B*

The lysine demethylase (KDM) 4 family of protein enzymes differ from FAD+ dependent protein enzymes in that they contain a JmjC-domain that requires Fe^+^, O_2_ and 2-oxogluterate as cofactors to properly function [28]. This family of proteins have the ability to demethylate di- and tri- methyl histone 3 lysine 9 and 36 (H3K9 and H3K36, respectively) as well as histone 1.4 lysine 26 (H1.4K26) and trimethyl histone 3 lysine 56 (H3K56) [28]. It has been demonstrated that large quantities of KDM4B protein in areas of inflammatory infiltrate increases the number of osteoclasts present [28]. Additionally, in osteoclasts, it has been shown that the inhibition of KDM4B in pre-osteoclasts results in reduced osteoclastogenesis [28]. Furthermore, with the inhibition of KDM4B, gene transcription of pro-inflammatory cytokines is also reduced [28]. Therefore, this data demonstrates that KDM4B is a transcriptional activator of osteoclast differentiation [28].

#### 2.3.3. *Jmjd3*

Similar to KDM4B, jumonji domain-containing 3 (Jmjd3 or KDM6B) is also a histone demethylase that contains a jumonji domain and has been shown to target trimethylation of histone 3 lysine 27 [29]. It has been shown that Jmjd3 is induced in bone marrow macrophages and plays an important role in RANKL-induced osteoclastogenesis [29]. Knockdown of the *Jmjd3* gene has been shown to reduce the amount of H3K27me3 demethylation at the transcriptional start site of *Nfatc1* and therefore causes reduced RANKL-induced osteoclastogenesis [29]. This suggests that activation of *Nfatc1* gene transcription requires the demethylation of H3K27me3 by JMJD3 and without it, osteoclast differentiation is attenuated [29].

### 2.4. Histone Deacetylases

**Figure 3:**
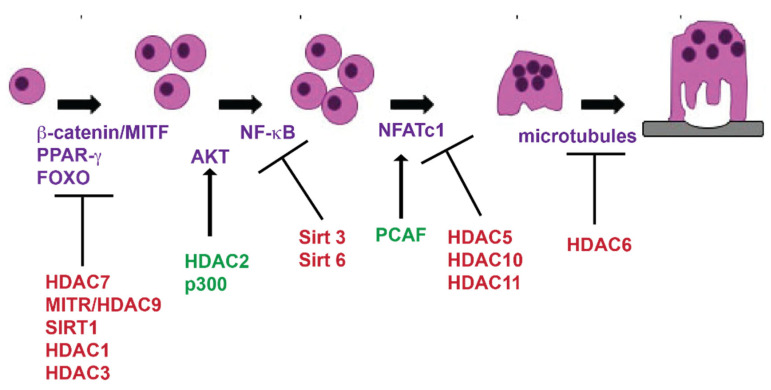
Histone deacetylases (HDACs) and histone acetyltransferases (HATs) expressed during osteoclast differentiation. Cartoon of osteoclast differentiation indicating the expression of HDACs and HATs. HDACs in red inhibit osteoclast differentiation and HDACs and HATs in green promote osteoclast differentiation. Proteins in purple are known targets of HDACs or HATs.

Like histone demethylases, histone deacetylases (HDACs), are eraser proteins. Their main function is to remove acetyl groups from either histone residues or transcription factors to repress gene expression [30]. There are 18 known HDACs within the human genome, each of which are distributed into 4 different classes: class I HDACs (HDACs 1,2,3,8), class II HDACs (HDACs 4,5,6,7,9,10), class III HDACs (Sirtuins 1–7) and class IV HDACs (HDAC11) [30]. They are divided based on their structure, enzymatic activity, location within the cell and their sequence homology to yeast [30]. While class I and III HDACs typically act like traditional deacetylases targeting histone residues, class II HDACs have been shown to function primarily independent of their deacetylation activity [31]. Figure 3 summarizes known activities of HDACs and HATs during osteoclast differentiation.

#### 2.4.1. *Hdac1*

HDAC1 is a transcriptional repressor that is present early during osteoclast differentiation [32]. It is expressed in osteoclast precursors but is reduced significantly after RANKL stimulation [32]. The main role of HDAC1 in osteoclasts is to act as a co-repressor [33]. HDAC1 is recruited to the promoter regions of genes like *Nfatc1* and *Oscar* to inhibit their expression [33].

#### 2.4.2. *Hdac2*

Opposite of HDAC1, HDAC2 expression increases during osteoclast differentiation [34]. Studies show that knock down of HDAC2 in osteoclasts not only inhibits osteoclast differentiation, but also prevents actin ring formation, fusion and osteoclast activity [34]. It is proposed that HDAC2 promotes osteoclast differentiation by activating AKT, which phosphorylates and deactivates FOXO1 as a negative regulator of osteoclast differentiation [34].

#### 2.4.3. *Hdac3*

HDAC3 is expressed in osteoclast precursors, however, it has relatively low expression during differentiation [35]. Knockdown of HDAC3 results in the inhibition of osteoclast differentiation as well as the osteoclast marker genes *Nfatc1*, *Ctsk* and *Dc-stamp* [36].

#### 2.4.4. *Hdac4*

HDAC4 is expressed in osteoclast precursor cells and decreases in expression as osteoclasts progress through differentiation [37]. Additionally, knockdown of HDAC4 in osteoclasts results in enhanced differentiation and upregulation of osteoclast genes [37]. More focused analysis by the Mansky lab is currently being performed to determine the role and significance of HDAC4 in vivo during osteoclast differentiation and skeletal modeling and remodeling.

#### 2.4.5. *Hdac5*

HDAC5 expression increases with RANKL stimulation of bone marrow macrophages. HDAC5 global knockout mice are osteopenic as HDAC5 affects bone formation through actions in osteoblasts and indirectly influences in vivo osteoclast function through effects in osteocytes. The in vivo role of HDAC5 in regulating osteoclast differentiation has not been determined; however, it is an area that the Mansky lab is actively investigating [38,39]. Studies with shRNAs targeting HDAC5 resulted in enhanced osteoclast differentiation and activity [37]. PCAF has been shown to acetylate NFATc1 the master regulator of osteoclast differentiation enhancing its stability. Overexpression of HDAC5 reduces PCAF acetylation of NFATc1. This data suggests that HDAC5 may play a role in regulating NFATc1 activity and thereby affects osteoclast differentiation [25].

#### 2.4.6. *Hdac6*

HDAC6 expression peaks during osteoclast fusion and is expressed abundantly in the cytoplasm [37]. Interestingly, knockdown of HDAC6 in osteoclasts does not seem to have a significant effect on osteoclast differentiation [37]. However, research shows that HDAC6 plays an important role in destabilizing the osteoclast cytoskeleton and inhibiting osteoclast migration and podosome formation [40,41].

#### 2.4.7. *Hdac7*

HDAC7 has been shown to be expressed early during osteoclast differentiation with continuously low levels of expression throughout [37]. Conditional knockdown of HDAC7 using *LysM-Cre*, that targets monocytes and myeloid lineage cells which includes osteoclasts, was found to enhance osteoclast differentiation and resulted in an osteopenic skeletal phenotype [31,42]. Besides MITF, HDAC7 was shown to inhibit β-catenin activity via NFATc1 and cyclin D1 expression in the presence of RANKL [42]. These studies suggest that HDAC7 is a negative regulator of osteoclastogenesis.

#### 2.4.8. *Hdac9*

Expression of HDAC9 in osteoclasts is detectable immediately following RANKL stimulation and then is significant reduced throughout the rest of differentiation [37]. HDAC9 was found to take part in a negative regulatory circuit with PPARγ and RANKL signaling. Both PPARγ and RANKL can inhibit *Hdac9* mRNA expression levels, while HDAC9 forms a complex with NCoR and SMART to inhibit PPARγ activity [32].

#### 2.4.9. *Sirt1*

Sirtuin 1 (SIRT1) is a repressor of osteoclast differentiation and activity by inhibiting RANKL signaling [43]. SIRT1 is able to do this by deacetylating and activating a group of inhibitors of osteoclastogenesis known as forkhead box proteins (FOXO) [43]. It has been shown that the loss of SIRT1 resulted in enhanced osteoclast differentiation and resorption due to increased acetylation of FOXO proteins [43].

#### 2.4.10. *Sirt3*

In osteoclasts, Sirtuin 3 (SIRT3) expression is induced by RANKL stimulation [44]. Loss of SIRT3 in osteoclasts results in an osteopenic bone phenotype in mice due to an increase in osteoclast number [44]. Additionally, it has been shown that this loss of SIRT3 resulted in an increase in the osteoclast gene markers *Oscar*, *Nfatc1* and *Atp6v0d2* [44]. This indicates that SIRT3 is a negative regulator of osteoclast differentiation [44].

#### 2.4.11. *Sirt6*

Sirtuin 6 (SIRT6) is expressed early during osteoclastogenesis once monocytes are stimulated with M-CSF and RANKL [45]. SIRT6 acts as a transcriptional repressor through inhibiting NF-κB transcription [45]. This inhibition results from the deacetylation of H3K9 on the promotors of target genes for NF-κB [45].

### 2.5. Reader Domain Containing Proteins

There are four subcategories of “reader” domain proteins. These are the proteins that recognize the modifications on the histones. The four domains are bromodomains, chromodomains, MBT domains and tudor domains. While there are multiple groups of proteins, very little is known about the functions of reader proteins in osteoclasts besides the bromodomain containing proteins.

#### Bromodomain Containing BET Proteins

Bromo and extra terminal (BET) proteins recognize acetylated histones. The inhibitor I-BET151 targets bromo and BET proteins. I-BET151 inhibits bone loss due to TNF-α induced inflammatory osteolysis, inflammatory arthritis and OVX [46]. Brd4 has been shown to interact and bind with PU.1 in an interdependent manner to regulate many of the key transcription factors that are essential for osteoclastogenesis and resorption [47]. 1-BET151 prevents expression of *Nfatc1* the master regulator of osteoclast differentiation [46]. Activity of the Brd4 inhibitor Jq1 is discussed in “Osteoporosis and Epigenetics”.

### 2.6. Conclusions

As is evident by the information contained in the above sections, there is mechanistic information known about the role of histone modifying proteins in regulating osteoclast differentiation; however, the information is limited due to the fact that most of these studies were done in cell culture. In recent years, there have been a few studies characterizing the histone modifications that occur during osteoclast differentiation; however, studies analyzing interactions between multiple epigenetic regulators are still scarce [47,48,49,50,51].

## 3. Epigenetic Control Besides Histone Modifications

Beside modifications to histones, modifications are made directly to nucleic acids to regulate gene expression. DNA methylation is a reversible modification that is made to the 5′-carbon of the cytosine residue. Generally, methylation configures DNA in a repressive state due to maintaining chromatin in a condensed state. In addition to DNA methylation, this section will also discuss methylation of adenosine residues in RNA and non-coding RNAs including micro-coding RNAs and long non-coding RNAs. Methylation of RNA and non-coding RNAs are additional epigenetic mechanisms used to regulate gene expression. Section three will discuss what is known about these epigenetic mechanisms and osteoclast differentiation.

### 3.1. Methylation

Methylation of cytosine is a highly conserved DNA modification. A recent study demonstrated that the promoter for *dendritic seven transmembrane protein* (*Dc-stamp* or *TM7SF4*) becomes less methylated as a women age [52]. Expression of *Dc-stamp* has been shown to be necessary for fusion of osteoclast precursors into multinuclear cells [53]. The authors conclude that monocytes from which osteoclasts are derived become reprogrammed through changes in methylation as a woman ages, accounting for the increase in bone resorption [52].

DNA methyltransferase 3a (DNMT3a) is a methyltransferase induced by RANKL stimulation and acts as a repressor of anti-osteoclastic genes there by promoting osteoclast differentiation [54]. Mice that are null for DNMT3a in osteoclasts have decreased number of osteoclasts and increased BV/TV compared to wild type littermates [54]. Mechanistically, DNMT3a suppresses *Irf8*, an inhibitor of osteoclast differentiation, by increasing methylation on distal regulatory elements [54]. Additionally, both DNMT3a and DNMT3b have been shown to interact and form a complex with the transcription factor, PU.1, to regulate PU.1′s target genes through DNA methylation [29].

### 3.2. Mettl3

N6-methyladenosine (m^6^A) is the most prevalent eukaryotic mRNA modification [55]. Methyltransferase-like 3 (METTL3) is partially responsible for the installment of m^6^A in humans [55]. It was the first component identified in the methyltransferase complex, and it functions as the catalytic core [55]. m^6^A and *Mettl3* expression has been shown to increase during osteoclast differentiation. Loss of *Mettl3* expression in either RAW 264.7 or BMMs results in larger osteoclasts with an increased number of nuclei per cell [55]. However, BMMs with reduced *Mettl3* expression did not demineralize calcium phosphate coated plates as well as control infected cells [55]. Interestingly BMMs with reduced *Mettl3* expression had reduced levels of *Dc-stamp* but increased levels of *Atp6v0d2* expression, suggesting a possible mechanism for the increased fusion measured [55].

### 3.3. Non-Coding RNAs

In addition to writer and eraser proteins, non-coding RNAs have been shown to have an effect on gene expression in many cell types. Transcripts from non-coding regions of the genome are termed non-coding RNAs. While these transcripts do not translate into a functional protein, recent studies have shown that they play an important role in biological processes such as cell growth, transcriptional regulation and tumorigenesis [56]. Additionally, these non-coding RNAs are expressed in a stage and cell specific manner. The major classes of non-coding RNAs are (1) micro RNAs (miRNAs) and (2) long non-coding RNAs (lncRNAs).

#### 3.3.1. Micro RNAs

Micro RNAs (miRNA) molecules are short approximately 25 nucleotide single-stranded, non-coding RNA molecules. miRNA bind to 3′UTR of mRNAs, resulting in their degradation or translational repression. There are over 2500 miRNAs known and approximately 60% of human mRNA molecules have miRNA binding sites [57]. miRNA sequences in the human genome are mostly encoded in intergenic or intronic regions. They are mostly regulated by the promoters that regulate protein coding genes but with alternative start sites [58]. Micro RNA (miR-148) is elevated in patients with osteoporosis and promotes osteoclast differentiation from monocytes [59]. miR-182 is highly expressed in TNF-α stimulated osteoclasts from mice that do not express RBP-J, a negative regulator of osteoclasts [60]. miR-182 promotes TNF-a enhanced osteoclast differentiation by regulating *Foxo3* and *Maml1* [60]. A more complete list of miRNAs that have been shown to play a role in regulating osteoclast differentiation are described in a recent review by Bellavia et al. [61].

#### 3.3.2. Long Non-Coding RNAs

Long non-coding RNAs (lncRNA) are functional RNAs that contain 200 nucleotides that are involved in transcriptional and posttranscriptional regulation of gene expression. DANCR is the first lncRNA identified as a potential biomarker for postmenopausal osteoporosis. DANCR overexpression in monocytes resulted in an increased expression of IL-6 and TNF-α. Both IL-6 and TNF-α promote bone resorption, and the positive correlation of DANCR with serum levels of IL-6 and TNF-α in osteoporosis patients suggest the involvement of DANCR in the pathology of osteoporosis [56].

## 4. Osteoporosis and Epigenetics

Despite efforts to provide early diagnosis of osteoporosis, fracture is often the first clinical sign of osteoporosis [62,63]. Fractures due to osteoporosis are estimated to be approximately two million per year [64]. Beside affecting lifestyle, fractures can affect a patient’s survival due to complications of a hospital stay [65]. Guidelines for treating osteoporosis include pharmacological intervention of both drugs to prevent resorption and stimulate bone formation, and adequate calcium and vitamin D to maintain or increase bone strength [63]. It has been suggested that up to 80% of the influences on bone mineral density are attributable to genetic factors. Using advanced techniques, multiple potential genetic markers of bone mineral density have been identified [66]. Recent data suggests that epigenetic proteins have a significant role in regulating osteoclast differentiation and activity. Drugs are being developed for the treatment and preventing bone loss. Recent studies have demonstrated a key role of DNA methylation in bone loss prevention [54]. Another example is the SIRT1 activator resveratrol, which promoted bone formation in a mouse model and represents a possible target for treating osteoporosis [67,68]. Inhibiting BRD protein BET with the specific inhibitor Jq1 inhibited osteoclast differentiation and restored bone loss [69]. Lastly, a recent study of human osteoclasts from female donors suggests that monocytes, the cells osteoclasts are derived from, are “reprogrammed” in vivo to remember age, menopausal and bone formation status. This “reprogramming” results in more bone resorption by osteoclasts and may be mediated through DNA methylation [52].

## 5. Aging and Epigenetics

While osteoporosis is often thought of as a disease that primarily affects postmenopausal women, both men and women lose bone as they age. One hallmark of aging is epigenetic changes characterized by increases in histone H4K16 acetylation, H4K20 trimethylation, H3K4 trimethylation and decreased H3K9 methylation and H3K27 trimethylation [70,71]. SIRT1, 3 and 6 in other mammalian cells have shown to contribute to healthy aging [72,73]. For example, SIRT 6 has been shown to deacetylate H3K9 which regulates genomic stability, NF-*κ*B signaling and glucose homeostasis [74,75]. Overexpression of SIRT 3 has been shown to improve regenerative capacity of hematopoietic cells [76]. Epigenetic marks such as trimethylation of H3K9 and H4K20 can impinge on regulation of telomere length, which is another hallmark of aging [77,78,79]. Another aspect of aging is changes in transcriptional networks, including miRNAs which regulate changes in inflammatory, mitochondrial and lysosomal degradation pathways [80,81,82,83]. In other tissues, HDAC inhibitors have been shown to be protective against the effects of aging [84]. Given the role of class II HDACs in regulating osteoclast differentiation, it is not apparent whether HDAC inhibitors are protective against age induced bone loss or if class specific HDAC inhibitors will need to be developed. Given the evidence of the role of epigenetic proteins in aging of other cell types, their role in regulating osteoclast gene expression and differentiation during aging is an area of underdeveloped research.

## 6. Bisphosphonates and Epigenetics

One of the most common types of drugs prescribed to patients with bone diseases are bisphosphonates, specifically nitrogen-containing bisphosphonates such as alendronate [85]. Nitrogen containing bisphosphonates inhibit prenylation of the GTP binding protein Ras [86]. The molecular targets of bisphosphonates are enzymes of the mevalonate pathway or prenyl protein transferases such as farnesyl diphosphate synthase (FDPS), thereby affecting protein prenylation which ultimately leads to osteoclasts undergoing apoptosis [85,86]. The mevalonate pathway provides metabolites for post translational modifications which can result in changes in HDACs, microRNAs and DNA methyltransferases. It has been hypothesized that metabolic pathways and the resulting epigenetic changes are the result of changes in concentration of NADPH. These studies were not done with osteoclasts, so it remains to be seen if this mechanism holds true during treatment of patients with osteoporosis [86].

## 7. Conclusions

While there is quite a bit of information that has been discovered concerning the role of epigenetic proteins in regulating osteoclast differentiation, there are still several unanswered questions. Do epigenetic proteins play a role in regulating enhanced osteoclast activity measured in women after they undergo menopause? Is the bone loss seen with individuals as they age due to changes in activity of epigenetic regulators? Are those the same changes that occur in postmenopausal women? Understanding how epigenetic regulators regulate and interact with each other in osteoclasts during all stages of life may unravel new therapeutic targets to combat enhanced osteoclast activity and bone loss.
